# Effects of test item disclosure on medical licensing examination

**DOI:** 10.1007/s10459-017-9788-8

**Published:** 2017-07-31

**Authors:** Eunbae B. Yang, Myung Ae Lee, Yoon Soo Park

**Affiliations:** 1 Department of Medical Education, Yonsei University College of Medicine, Seoul, South Korea; 2 Korea Institute for Curriculum and Evaluation, Seoul, South Korea; 3 Department of Medical Education, College of Medicine at Chicago, University of Illinois, Chicago, IL USA

**Keywords:** Item disclosure, Medical licensing examination, Validity, Fairness

## Abstract

In 2012, the National Health Personnel Licensing Examination Board of Korea decided to publicly disclose all test items and answers to satisfy the test takers’ right to know and enhance the transparency of tests administered by the government. This study investigated the effects of item disclosure on the medical licensing examination (MLE), examining test taker performance, psychometric characteristics, and factors affecting pass rates. This paper analyzed examinee performance data (*n* = 20,455) from 41 medical schools who took the MLE before (2009–2011) and after (2012–2014) the item disclosure policy (5548 total items). Changes in passing rates, performance of examinee, difficulty and reliability of the test, and factors affecting pass rate of the medical licensing examination before and after item disclosure were analyzed. In order to identify changes caused by item disclosure in the effects of student and school variables on the passing rate of MLE, Binary Logistic Hierarchical Linear Model was used. There was no significant change in pass rates before and after item disclosure. There was a modest increase in the proportion of test takers in the high-scoring group, following item disclosure. Degree completion status, gender, age of applicants and school mean were significant factors affecting pass rates, regardless of item disclosure. There was no difference between passing rates before and after item disclosure with respect to student- and school-level variables. Despite potential concerns for changes in test and examinee characteristics, empirical findings indicate that there was no significant difference caused by implementing item disclosure.

## Introduction

Medical licensing examination is a selection process that identifies qualified candidates and grants them license to practice medicine. In this paper, we refer to the general term medical licensing examination (MLE) as a high-stakes examination, administered at a national level for medical licensing purposes. The agency administering MLEs typically maintains item banking systems, enforces test security measures, and has policies against *item disclosure*—publicly releasing all exam questions, answer keys, and examinee performance data following exam administration. Maintaining the security of test items and scores is paramount if tests are to provide useful inferences on examinee performance (Cohen and Wollack 2006). However, publicly disclosing item-level information has historically faced challenges on grounds of fairness and validity (Bersoff 1981; Camilli 2006; Park and Yang 2015). Proponents of item disclosure emphasize the need for test takers to know the actual test items and answer keys in addition to other relevant performance data, based on the principle of fairness and public accountability. They assert that testing is a type of social policy, which deserves thorough public scrutiny and discussion. On the other hand, opponents state that the security of testing already support fairness, and thus, disclosure of test items may undermine the validity of the test; for example, exposure of reused items may inflate the equated test scores (Gilmer 1989). Opponents also emphasize the burden in costs that may be accompanied by each new cycle of test development if full item disclosure were to be implemented.

The discussion of item disclosure can be divided into regulations enforced by legislative bodies and empirical research studies. In the United States, laws dictating item disclosure date back to the 1970s, with multiple federal and state-level regulatory proposals, in what are known as *Truth*-*in Testing* (TiT) laws. While only state-level regulations on item disclosure of post-secondary school entrance examinations have passed in California in 1978 and New York in 1979 (Dario 1980), these legislative actions have increased public awareness and raised issues in the testing community on the role and impact of item disclosure (Bersoff 1981; Bower 1987; Florio 1979; Greer 1984). To date, several studies have evaluated the influence of test item disclosure, analyzing item difficulty, reuse of items, and changes in setting cut-off levels. Veerkamp noted that item exposure has received much attention in the assessment studies and preserving the quality in the item pool requires controlling item exposure (Veerkamp and Glas 2000). On the other hand, Wood et al. concluded that the exposure of prior test items had little impact on the examinee’s performance who took the Medical Council of Canada Evaluation Examination (Wood 2009; Wood et al. 2010). Other researchers reported that there were some increase in student scores on reused recall-type items but did not result in increased overall scores (Wagner-Menghin et al. 2013); moreover, there were findings that also report minimal impact of item disclosure or reusing items (Stricker 1984). As such, research conclusions vary on the impact of test item disclosure and until now, such discussions have not reached consensus.

In South Korea, discussions on disclosing test items of the MLE began in 2011. The National Health Personnel Licensing Examination Board (NHPLEB),^1^ the testing authority that administers the MLE, decided that beginning in 2012, test items and answers will be made publicly available to satisfy the test takers’ right to know and enhance the transparency of tests administered by the government. This policy change provides a natural quasi-experiment design allowing an investigation into the impact of item disclosure.

This study addresses two research questions: (1) how did the implementation of item disclosure policy affect test performance and psychometric characteristics of the MLE test forms and (2) are factors contributing to pass-fail status consistent following the implementation of the item disclosure policy? In particular, this paper examines the effect of item disclosure of the MLE, by comparing test characteristics, such as passing rate, item characteristics and reliability, changes of examinee test score, and influence of applicants and school variables to pass the MLE before and after policy implementation.

## Methods

### Data

The MLE administered by the NHPLEB consists of a written test and a clinical skills examination. Passing both the written and clinical skills component of the MLE is a requirement to practice medicine in South Korea, and examinees are generally composed of medical students (final year of training and within 6 months of graduating with a medical science degree) or recent graduates. This study focuses on multiple-choice items used in the written test. A total of 5548 test items were analyzed, consisting of three test domains: (1) Introduction to Medicine (1272 items; 23%), (2) Specialized Topics in Medicine (4080 items; 73%), and (3) Public Health and Medicine Law (196 items; 4%). All items were multiple-choice questions in the selected-response format (single-best answer). The MLE in administered one each year and uses a fixed passing score of 60%, regardless of year-to-year variations in test difficulty; that is, as the number of applicants who scored over 60% increases, the passing rate would also increase. While there were reused items pulled from an item bank during years prior to item disclosure (Year 2009, 2010, and 2011 in the study data), there were no reused items in subsequent exams after implementation of the item disclosure policy (Years 2012, 2013, and 2014 in the study data). All items administered after the item disclosure policy were available to the public, preventing them to be reused in subsequent exams.

Data were extracted from the archived database of NHPLEB of Korea. The MLE including the data had been administered to 20,455 (13,172 males, 7283 females) enrolled medical students and graduates from 41 medical schools from 2009 to 2014. The 15,696 test takers were in their 20 s (76.7%) and the remaining 4759 were in other age groups (23.3%). Applicants included 19,416 candidates for bachelor’s degree of medicine and 1039 bachelor’s degree holders of medicine (5.1%). The applicants were divided into two groups: (1) examinees who took South Korea’s MLE from 2009 through 2011 prior to item disclosure and (2) the other group of examinees who took the exam from 2012 through 2014 after the item disclosure.

### Analysis

Data were analyzed to examine changes in passing rates, difference between passing and mean score, difficulty and reliability by year and the change in applicants’ test scores in order to investigate the impact posed by item disclosure. The number of test items and the scores assigned to each item varied each year (see Table 1). And as such, the test forms could not be equated, and we used performance levels of applicants using linear transformation, converting raw scores into *T*-scores with a mean of 50 and a standard variation of 10. In addition, to examine possible differences in percent scores and pass-fail status before and after the item disclosure policy, we ran random-intercept regression models, with item disclosure coded as a binary predictor and test year specified as random intercept. Stata 14 (College Station, TX) was used in data compilation and analysis.

**Table 1:** Passing rate, mean percent score, and reliability of the medical licensing examination

Category	Before item disclosure			After item disclosure		
	2009	2010	2011	2012	2013	2014
Number of examinees	3750	3452	3236	3363	3177	3287
Pass rate (%)	93.60	97.02	94.44	96.91	96.22	96.65
Number of items	550	500	500	450	400	400
Reliability (Cronbach’s alpha)	.96	.96	.96	.95	.95	.94
Percent Score: Mean	72.53	77.89	74.09	76.83	75.59	75.32
Percent Score: SD	9.48	8.08	8.03	7.81	7.88	7.37

In order to identify changes in the effects of student and school variables on the passing rate of the MLE, due to item disclosure, Binary Logistic Hierarchical Linear Model (BLHLM) was used (Skrondal and Rabe-Hesketh 2004). This model is a type of Generalized Hierarchical Linear Model used when the dependent variable is binary. The dependent variable in this paper was the dichotomous classification of “passed or not passed” (Not passing = 0, Passing = 1), and the predictor variables were gender of applicants (male = 0, female = 1), degree completion status (Expected graduate = 0, Graduate = 1), age (in their twenties = 1, thirties = 2, forties = 3, fifties = 4, sixties = 5), and school location (capital area = 1, others = 0). In the analysis, school mean was added as a variable to control the scores of students. Variables except school mean (average school-level pass rate) are categorical variables and thus used as they were, while non-categorical variable of school mean was adjusted by grand mean centering. For the binary logistic multilevel analysis, HLM 6.0 was used. The BLHLM employed in this paper was as follows, where Eq. (1) shows the examinee-level effects and Eq. (2) shows the school-level effects associated with the multilevel model:

1 $$\log \left(\frac{{p_{ij} }}{{1 - p_{ij} }}\right) = \eta_{ij} = \beta_{0j} + \beta_{1j} X_{1ij} (Degree) + \beta_{2j} X_{2ij} (Gender) + \beta_{3j} X_{3ij} (Age)$$

2 $$\beta_{0j} = \gamma_{00} + \gamma_{01} (School\_Mean)_{j} + \gamma_{02} (School\_Location)_{j} + u_{0j} ,_{{}} u_{0j} \sim N(0,\tau_{00} )$$

Following standard BLHLM interpretation, the parameter *β*_0*j*_ indicates the average log-odds of school *j*; *γ*_00_ indicates the average log-odds of all schools; and *γ*_0*q*_ indicates the log-odds effect of the *q*th school-level variable. In addition, the parameter *u*_0*j*_ and the parameter *τ*_00_ indicate school *j*’s effect and the variance of school effects, respectively.

This study was approved by the Institutional Review Board at Severance Hospital of Korea (4-2015-0064).

## Results

### Changes in the passing rate and performance level of the MLE applicants

Changes in passing rates of the MLE applicants are presented by year (before item disclosure: 2009–2011; after item disclosure: 2012–2014) in Table 1. On average, there was a modest increase in the passing rate by 1.6% between the periods before (95.0%) and after item disclosure (96.6%). Performance in Year 2010 was highest (Mean % Score = 78, SD % Score = 8; Pass Rate = 97%); the lowest performance was in 2009 (Mean % Score = 73, SD % Score = 9; Pass Rate = 94%). However, there were no consistent trends between years.

**Fig. 1:**
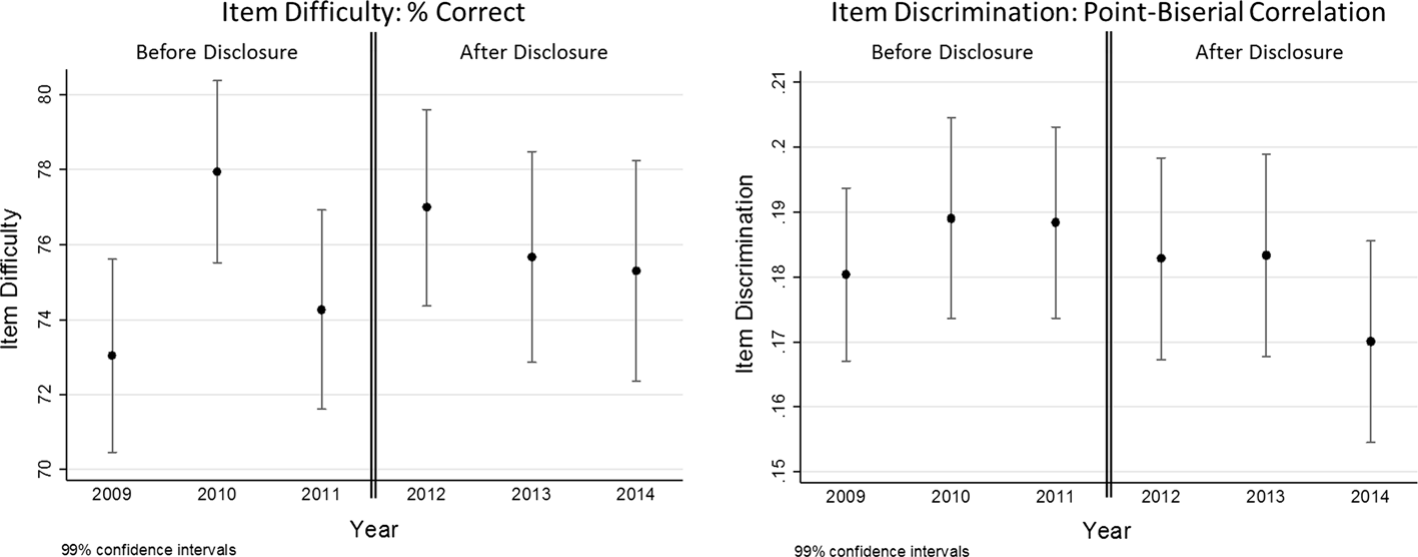
Item characteristics by year: item difficulty (% correct) and item discrimination (point-biserial correlation) *values* represent mean value ± 99% confidence interval, to allow multiple-group comparison between years based on Bonferroni correction

There were no significant differences in overall item difficulty (% correct) before and after disclosure (Before: Mean Difficulty = 75, SD = 23; After: Mean Difficulty = 76, SD = 22; *p* = .233). Likewise, there were no significant differences in overall item discrimination (point-biserial correlation) between items administered before and after item disclosure (Before: Mean Discrimination = .19, SD = .13; After: Mean Discrimination = .18, SD = .12; *p* = .150). Figure 1 shows the item characteristics (difficulty and discrimination) by test administration year. The reliability (Cronbach’s alpha) was consistent during the periods (range = .94–.96).

The converted raw scores of examinees into *T*-scores by year and the distribution of *T*-scores are shown in Table 2. After item disclosure in 2012, the percentage of the high scoring group with standard score of 60 or more increased, and the standard score of the 40–50 points section generally rose every year during years following item disclosure (2009, 2010, 2011). In the standard score of other sections, there were no meaningful trends observed before and after item disclosure.

**Table 2:** *T*-score distributions of the medical licensing examination applicants (unit:  %)

*T*-score range	Before item disclosure			After item disclosure			Total
	2009	2010	2011	2012	2013	2014	
<40	10.42	14.72	14.22	14.96	14	12.99	13.46
40–50	30.24	27.55	30.47	30.13	31.73	32.83	30.45
50–60	51.25	44.18	41.34	40.32	39.61	39.82	43.05
>60	8.09	13.56	13.97	14.6	14.66	14.36	13.05

*T*-Score range identified as “<40” (2 SDs or more below the mean), “40–50” (<1 SD below mean), “50–60” (more than 1 SD above mean), and “>60” (2 SDs or more above mean)

In addition, the impact of item disclosure on performance (% score and pass-fail rates) were examined, taking into account differences in test year. Overall, results show that there were no significant differences in % score, *p* = .426. However, there was modest variability in % scores by year. Likewise, there was no significant difference in pass-fail status before and after item disclosure, *p* = .388. For pass-fail status, test was minimal variability between years. Table 3 shows the random-intercept regression results.

**Table 3:** Impact of item disclosure on test score (% score) and pass-fail status: random-intercept regression and random-intercept logistic regression

Effect	Parameter	Percent scores: random-intercept regression			Pass rate (pass = 1, fail = 0): random-intercept logistic regression		
		Estimate (SE)	*p* value	95% CI	Odds ratio (SE)	*p* value	95% CI
Fixed	Item disclosure	1.08 (1.35)	.426	(–1.58, 3.73)	1.07 (.09)	.388	(.92, 1.26)
	Intercept	74.84 (.96)	<.001	(72.96, 76.72)	13.64 (.78)	<.001	(12.20, 15.25)
Random	Var (year)	2.73 (1.59)		(.87, 8.54)	.00 (.01)		(.00, .05)
	Var (residual)	66.86 (.68)		(65.54, 68.20)			
Log likelihood		–68500.19			–4726.95		

Item disclosure is coded “1” if data come from scores or pass-fail status following item disclosure policy (Years 2012, 2013, and 2014) and coded “0” if data come from scores of pass-fail status prior to item disclosure policy (Years 2009, 2010, and 2011). Model estimation based on maximum likelihood

### Changes in the effects of variables affecting passing rate

Table 4 shows the results of the BLHLM. The BLHLM allows specifying examinee- and school-level predictors to the model to examine factors affecting pass rates by year. The examinee-level results showed that the degree completion status, gender, and age were significant predictors of pass rates for all years. The degree completion status had a negative regression coefficient (and odds ratio <1), suggesting that applicants who are expected to earn a bachelor’s degree are more likely to pass the exam. Gender had a positive regression coefficient with odds ratio >2, meaning that the odds of female students passing the exam were more than twice higher male students. Age had a negative regression coefficient and the odds ratio was <1, indicating that younger applicants were more likely to pass the exam. With respect to the level of school, the higher school mean indicates a higher passing rate, but this was not statistically significant in 2010. The regression coefficients of school location were negative, except in 2011, which showed that the passing rate of students in capital area tends to be lower, but this was not statistically significant, except in 2009. There was no differences between the passing rates before and after item disclosure with respect to variables relating to students and schools.

**Table 4:** Influence of applicant and school-level factors on pass rates on the medical licensing examination: Binary Logistic Hierarchical Linear Model (BLHLM)

Item disclosure	Factors		*B* (SE)	Odds ratio	*B* (SE)	Odds ratio	*B* (SE)	Odds ratio
Before item disclosure	Year		2009		2010		2011	
	Level 1: examinee	Degree	−2.95 (.26)**	.05	−2.57 (.25)**	.08	−2.63 (.27)**	.07
		Gender	.86 (.17)**	2.36	.76 (.15)**	2.14	1.13 (.20)**	3.10
		Age	−.93 (.20)**	.40	−.94 (.15)**	.39	−.40 (.20)*	.67
	Level 2: schools	Location	−.52 (.16)**	.59	−.42 (.23)	.66	.09 (.22)	1.09
		Mean	.03 (.01)*	1.03	.00 (.01)	1.00	.02 (.01)**	1.02
After item disclosure	Year		2012		2013		2014	
	Level 1: applicants	Degree	−1.69 (.25)**	.19	−2.12 (.23)**	.12	−2.39 (.24)**	.09
		Gender	1.00 (.26)**	2.72	1.08 (.16)**	2.95	.89 (.16)**	2.44
		Age	−1.16 (.17)**	.32	−.65 (.18)**	.52	−1.01 (.15)**	.36
	Level 2: schools	Location	−.38 (.26)	.68	−.21 (.21)	.79	−.38 (.27)	.68
		Mean	.02 (.01)**	1.02	.03 (.01)**	1.03	.03 (.01)**	1.03

Significance level denoted by * *p* < .05 and ** *p* < .01

Degree 0 = expected graduate, 1 = graduate; Gender 0 = male, 1 = female; Age 1 = twenties, 2 = thirties, 3 = forties, 4 = fifties, 5 = sixties; Location 1 = capital area, 0 = others; Mean was adjusted by grand mean centering

## Discussion

Following the implementation of the item disclosure policy, the MLE in South Korea began releasing all test items, answers, and performance data to the public. This study investigated whether the disclosure of test items impacted passing rate, average scores before and after the item disclosure, and factors that influence pass rates. Overall, the results did not show meaningful changes in the passing rate before and after item disclosure. While, passing rates in the high-scoring group and a specific score section rose slightly, the overall change in pass rates was modest at 1.6%. As for the effects of variables affecting the passing rate of the MLE, regardless of the item disclosure policy, degree completion status, gender, and age of students were significant predictors, and the school mean had statistical significance in terms of school-level variable. As such, this research was unable to identify any meaningful change brought about by item disclosure in MLE. These findings are consistent with previous research by Stricker (1984), which concluded that following test item disclosure of Scholastic Aptitude Test of the United States, there were no significant changes or effects on the applicants’ performance. Likewise, Wood (2009) also found no change in test characteristics and that repeat examinees do not appear to be advantaged by encountering reused question. It is worth noting that prior computer simulation studies by Gilmer (1989) in the measurement literature revealed that item disclosure could possibly lead to the passing unqualified examinees and bring about an approximately 10% increase of passing rate as compared with the time of non-disclosure. However, this study provides empirical findings based on actual data motivated by a national policy change, which has allowed a natural quasi experiment to examine the impact of item disclosure in large-scale high-stakes testing conditions.

The finding that there was not any noticeable change in the function of the MLE before and after item disclosure provides several implications. First, the MLE uses a fixed passing standard, where all applicants who scored above 60% of total score points, regardless of the difficulty of test items, will successfully pass the exam. Establishing such a passing score involves risks. That is, depending on the difficulty of each session, the number of successful applicants can vary. In other words, if item difficulty is set much higher or lower than previous years, the validity of the reference score of the examination may be undermined. In order to minimize this risk, the NHPLEB of Korea included items with correct rate of over 80% which accounted for more than half of all items each year. This trend of difficulty of the MLE is verified by the fact that there was no difference in the item difficulty before and after item disclosure. For this reason, it seems that despite item disclosure, there was nearly no change in the passing rate and the performance level of applicants. Second, since establishment of a test item bank of the MLE in the NHPLEB of Korea, it holds items 20 times as large as the number of actual test items, and continues to develop new items every year. Even after the NHPLEB implemented item disclosure, the maintenance of item bank and development of new items continued, and the test items of the MLE were selected mostly from the item bank. As the items stored in the test item bank carry proven level of item difficulty and item discrimination, many countries adopt this test bank methodology to maintain the stability of testing. After item disclosure, strategy of increasing the amount of new items in the item bank can deal with the issues on test validity (Wagner-Menghin et al. 2013). Accordingly, it can be inferred that, by continuing to maintain and make good use of test item bank even after the item disclosure, the impact of item disclosure policy on the MLE was minimized.

It should also be noted that students were already practicing with the prior test items of the MLE as important study material, even before the official item disclosure. While this violates test policy, it was found that after an MLE test administration, students restored test items by memory and shared them with others. When questions are passed down from one student to another, it creates a potential unfair advantage as some students have access to the items while others do not (Kim 2013). In addition, it is common for students to regard passing exam content to fellow students and using passed content for studying (Wagner-Menghin et al. 2013).

There are some limitations to this study. While this study presents large-scale data of examinees across six test administration years (on average of 3409 examinees per year), we cannot claim equivalence of examinee ability between test years. Moreover, we were unable to identify reused items for possible equating or trended analyses of item analyses across test years. Finally, the passing score applied in the analysis is based on a fixed 60% cutscore following the MLE testing policy, which is not criterion based. Efforts are underway to examine uses of criterion-based standards in future test administrations and also to study differences in the impact of item disclosure based on examinee subgroup analyses.

In summary, our empirical results derived from large-scale and longitudinal national data show that there were no meaningful differences in examinee performance, psychometric characteristics, and factors contributing to MLE pass rates following the item disclosure policy. These findings provide some reassurance for NHPLEB of Korea, which decided to disclose all test items and answer keys to students to promote needs for fair and transparent testing environment.

## Footnotes

1 National Health Personnel Licensing Examination Board URL: http://www.kuksiwon.or.kr/EngHome/Index.aspx.
